# Cuticular hydrocarbons correlate with queen reproductive status in native and invasive Argentine ants (*Linepithema humile*, Mayr)

**DOI:** 10.1371/journal.pone.0193115

**Published:** 2018-02-22

**Authors:** Sílvia Abril, Mireia Diaz, Alain Lenoir, Carolina Ivon Paris, Raphaël Boulay, Crisanto Gómez

**Affiliations:** 1 Departament de Ciències Ambientals, Universitat de Girona, Girona, Spain; 2 Institut de Recherche sur la Biologie de l’Insecte, Université François Rabelais de Tours, Tours, France; 3 Departamento Ecología, Genética y Evolución, Facultad de Ciencias Exactas y Naturales, Universidad de Buenos Aires, Buenos Aires, Argentina; Universidade de Sao Paulo Faculdade de Filosofia Ciencias e Letras de Ribeirao Preto, BRAZIL

## Abstract

In insect societies, chemical communication plays an important role in colony reproduction and individual social status. Many studies have indicated that cuticular hydrocarbons (CHCs) are the main chemical compounds encoding reproductive status. However, these studies have largely focused on queenless or monogynous species whose workers are capable of egg laying and have mainly explored the mechanisms underlying queen-worker or worker-worker reproductive conflicts. Less is known about what occurs in highly polygynous ant species with permanently sterile workers. Here, we used the Argentine ant as a model to examine the role of CHCs in communicating reproductive information in such insect societies. The Argentine ant is unicolonial, highly polygynous, and polydomous. We identified several CHCs whose presence and levels were correlated with queen age, reproductive status, and fertility. Our results also provide new insights into queen executions in the Argentine ant, a distinctive feature displayed by this species in its introduced range. Each spring, just before new sexuals appear, workers eliminate up to 90% of the mated queens in their colonies. We discovered that queens that survived execution had different CHC profiles from queens present before and during execution. More specifically, levels of some CHCs were higher in the survivors, suggesting that workers could eliminate queens based on their chemical profiles. In addition, queen CHC profiles differed based on season and species range (native vs. introduced). Overall, the results of this study provide new evidence that CHCs serve as queen signals and do more than just regulate worker reproduction.

## Introduction

Social insects are good models for studying chemical communication since their striking self-organisation and division of labour involve sophisticated chemical systems. Maintaining social cohesion and resolving potential reproductive conflicts between queens and workers is possible because nestmates efficiently exchange information on the physiological state of their reproductive(s). According to Keller and Nonacs [1], queens produce honest fertility signals to which workers react by refraining from reproducing or preventing other workers from doing so (i.e., worker policing, sensu Ratnieks [2]). This system allows queens to maintain reproductive supremacy within the colony, while allowing workers to accurately assess queen health and fertility and then react as needed to increase colony reproductive efficiency and, consequently, their own inclusive fitness [1, 3, 4]. Because this strategy benefits both queens and workers, it is evolutionarily stable. It has been hypothesised that if these chemical compounds act as honest signals, they should be correlated with metrics of reproductive status and fertility [1, 3]. Many studies have indicated that cuticular hydrocarbons (CHCs) are the main queen signals—they encode information about queen reproductive status and fertility (see [3–7]). However, these studies have largely focused on queenless or monogynous species whose workers are capable of egg laying and have mainly explored the mechanisms underlying queen-worker or worker-worker conflicts. Less is known about what occurs in highly polygynous ant species with permanently sterile workers. In such species, how can workers manage the colony’s reproductive output when they have dozens of queens to care for? More interestingly, how is the situation handled in large polydomous colonies, which comprise thousands of individuals? Under these circumstances, information sharing between queens and workers should be highly efficient and involve chemically complex queen signals that allow workers to regulate queen fertility and increase colony reproduction [1].

The Argentine ant (*Linepithema humile*, Mayr) is a good model with which to examine how workers in such complex insect societies regulate queen-worker interactions. It is a major invasive species, and in its non-native range, it is unicolonial—the population consists of nests without clear colony boundaries—and highly polygynous [8, 9]. Its colonies are seasonally polydomous as a consequence of environmental conditions and food resource availability [10, 11]. Moreover, its workers are completely sterile [12].

In the Argentine ant’s native range, populations are also unicolonial, highly polygynous, and polydomous. It can form supercolonies just as in introduced populations [13]. The only difference is supercolony size and longevity. The largest Argentine ant supercolony observed to date is the main European supercolony, which stretches over more than 6,000 kilometres from Italy to Portugal [12]. Two other supercolonies have been identified in Europe: the Catalan and the Corsican supercolonies [14, 15, 16]. A recent study showed that the main European supercolony is consistently more aggressive, more efficient at exploring novel environments, and more effective at exploiting food resources than the Catalan supercolony and native supercolonies [17].

The seasonal life cycle of the Argentine ant has only been studied in its introduced range [18, 19]. From a reproductive perspective, it appears that queens experience three important annual physiological periods: maximum egg laying in the spring, renewed egg laying in the autumn, and a pause in the winter [18, 19]. However, this conclusion is based on indirect observations of the brood content of nests; the actual physiological state of queens is unknown. Another crucial moment is the annual execution period. Each May, workers execute up to 90% of their colony’s mated queens [20]. Afterwards, colony numbers bottom out for the year [21]. The execution of queens by workers is relatively rare in ants, and to our knowledge, the Argentine ant is the only ant species that exhibits such a striking mechanism of queen turnover year after year. It is unknown if this same behaviour is also present in the species’ native range.

Numerous studies on the Argentine ant have examined the effects of diet on worker CHC profiles [22–25], as well as the ability of worker and male CHCs to serve as nestmate recognition cues [16, 26–39]. However, only a few studies have focused on the role of CHCs as queen signals in this species [40, 41] and, to date, only one has examined how a queen’s physiological state affects her CHC profile [42]. De Biseau and colleagues [42] demonstrated that ovarian activity in Argentine ant queens is correlated with changes in CHCs, suggesting that species with permanently sterile workers also use chemical signals to convey information about queen reproductive status. The researchers stated that, since Argentine ant workers are sterile, the correlation between ovarian activity and CHC profiles may reflect the existence of forms of reproductive regulation beyond the repression of worker reproduction. For example, if workers can gauge queen fertility, they could favour more fertile queens and thus enhance colony reproductive output and their own inclusive fitness given that they are at least to some extent related to the queen they favour. [42]. The authors concluded that, to test this hypothesis, research would have to determine whether a correlation exists between reproductive performance and chemical signals in Argentine ant queens. They also underscored the need to assess whether workers execute queens based on differences in their CHCs. Such data could reveal unknown facets of reproductive regulation in this polygynous ant species.

In this study, we examined four key ideas: does variation in the CHC profiles of Argentine ant queens depend on 1) fertility and/or reproductive status, 2) season, and 3) range (i.e., native vs. introduced), and 4) do the profiles of queens that survive execution differ from those of queens who do not?

To address the first question, we investigated how ovarian activity and egg laying were related to the CHC profiles of Argentine ant queens. Although de Biseau and colleagues [42] had already demonstrated that ovarian activity is correlated with the CHC profiles in queens of this ant species, their results were mainly qualitative. In this paper, we explore this point more in depth by: 1) adding a new reproductive variable, the egg-laying rate, something non-explored before in this ant species in relation to the CHC profiles of queens, and 2) searching for certain CHCs correlated with fertility that could therefore signal reproductive information. Indeed, a correlation between CHCs and fertility in a species with permanently sterile workers, such as the Argentine ant, could reflect the existence of forms of reproduction regulation beyond the simple repression of worker reproductive activity.

Studies addressing seasonal variation in CHC profiles are relatively scarce, and little is known about CHC profiles in Argentine ant queens, especially in the species’ native range. Consequently, our second question helped reveal information about the seasonal dynamics of CHC profiles for queens in both native and introduced ranges. We also compared the CHC profiles of queens near the invasion front to those of queens far from the invasion front, given that such nests differ in queen size and number [43, 44].

In the Argentine ant, the CHC profiles of workers from the species’ introduced range are less diverse than those of workers from the species’ native range, likely as a consequence of a founder effect and subsequent range expansion associated with introduction [29, 45]. Our third question was focused on whether a similar difference was seen in queens from the native range versus the introduced range (i.e., in the main European supercolony) and, if so, whether these differences affected queen fertility signalling.

Finally, our last question deals with the relationship between CHC profiles and the probability of surviving execution. We examined this link by sampling queens in the field before, during, and after the execution period. If workers eliminate queens based on their CHCs, we would expect to see differences in queen CHC profiles before and after the execution period.

## Materials and methods

We are grateful to the authorities of the Administration of National Parks of Argentina (APN) for their permission to conduct our study in the Natural Reserve of Otamendi (project number: DCM 427). We would like to thank the Argentinian Ministry of the Environment and Sustainable Development for the exportation permits: 12353/2013 and 1614/2013.

### Ant collection and rearing

Argentine ant queens were collected by removing nests situated under stones in Argentina (native range) and Spain (introduced range). In Argentina, the queens were taken from seven nests in the Natural Reserve of Otamendi (34°14′03″S, 58°53′10″W; 1–10 m.a.s.l.), which is 70 km northeast of Buenos Aires. Sampling took place along the edges of the reserve’s main road, which links the railway station in the village of Otamendi to the Parana River. The road is flanked on either side by canals; between the road and the canals are strips of shrubland (2–6 m wide) containing grasses and scattered trees, which are intended to buffer flooding. In Spain, the queens were taken from twenty nests situated in two natural open cork oak secondary forests found on the southern edge of the Gavarres range (NE Iberian Peninsula) (41°49′24′′N, 3°00′18′′E; 100–150 m.a.s.l.) that have been invaded by the main European supercolony. This area was completely outside the limits of the Catalan supercolony. One sampling area was located near the village of Santa Cristina d’Aro, and the other was located in the Cadiretes mountains, near Pedralta. These areas are characterized by the presence of an invasion front. We defined two different zones according to their proximity to the invasion front: 1) the contact zone, where the Argentine ant and native ants are still in contact; and 2) the invaded zone, where the Argentine ant has almost completely excluded native ants [44, 46–47]. The contact and invaded zones of the two study areas were ±1 km apart and had similar environmental characteristics.

Queens were collected at these locations during the three important periods of their annual life cycle: 1) the winter, their hypothetical period of physiological rest (February 2013 in Spain, July 2013 in Argentina); 2) the spring, when their egg-laying rate is highest (April 2013 in Spain, October/November 2012 in Argentina); and 3) the autumn, their hypothetical period of renewed egg laying (October 2012 in Spain, April 2013 in Argentina). Further sampling took place during the spring in Spain to characterise the relationship between CHC profiles and queen executions. More specifically, queens were collected from the beginning to the end of the execution period. Thanks to previous research, we knew when we could expect to observe queen executions. In our period of study, they began in early May 2013: queens with amputated legs or gasters were observed in nests during the first week of May 2013. During the execution period, we estimated queen densities in nests on a weekly basis as per Abril et al. [21]. In brief, we estimated Argentine ant queen densities by taking samples of two liters of soil from nests in the field. Afterwards, we manually extracted the queens present in each sample, and calculated queen density per liter of nest soil. We assumed the execution period had ended when queen densities remained stable for two consecutive weeks, which occurred by the last week of May 2013. Thus, queens sampled that week were considered to be survivors. This sampling scheme allowed us to examine seasonal differences between queens from the native and introduced ranges (hereafter native and invasive queens, respectively) and between queens from the contact and invaded zones within the introduced range.

To study the relationship between fertility and CHC profiles in young queens of different reproductive status, female sexual pupae were collected in mid-May 2013 from a total of four nests belonging to the main European supercolony in the city of Girona (Catalonia, Spain) (41°59′27.62″N, 2°49′11″E; 64–65 m.a.s.l.). They were collected by digging nests, and once into the laboratory, they were manually separated from the soil and gently placed into artificial nests where they were reared until they emerged. Recently emerged queens were then sampled over the course of development. We looked specifically at five different groups: 1) 24-hour-old non-laying virgin queens; 2) 4-day-old non-laying virgin queens; 3) non-laying mated queens (24 hours after mating); 4) 2-week-old laying mated queens; and 5) 2-week-old laying virgin queens.

To obtain mated queens, virgin queens were placed in small arenas with 10 males and left there until they shed their wings. The dealated mated queens were isolated with a small group of workers until egg laying began (i.e., when they were approximately 2 weeks old). To obtain the dealated laying virgin queens, virgin queens were isolated with a small group of workers but no males until they shed their wings and started to lay haploid eggs (i.e., when they were approximately 2 weeks old).

### Fertility status

To examine the relationship between fertility and CHC profiles in queens, two reproductive variables were measured: the egg-laying rate, which is a good indicator of overall egg-laying activity, and the ovarian index (OI), which is a good indicator of reproductive investment [48].

Queen egg-laying rate was estimated only in the invasive queens. First, queens were placed in test tube nests with 3–5 workers for a 24-hour period. The test tubes (70 mm in length x 10 mm in diameter) were made of transparent plastic and had plastic lids. The inner side of each lid was covered by a layer of dry plaster of Paris, which was connected by a cotton wick to a small chamber filled with water. As a result, the inside of the tube remained permanently moist over the course of the observations. For the field-collected queens (n = 96), the test tube nests were kept at the mean soil temperature of their nests in nature (monitored using HOBO H8 Pro Series data loggers). For the laboratory-reared queens (n = 64), the test tube nests were kept at 28°C, which is the optimal temperature for egg laying in the Argentine ant [49]. After 24 hours, the number of eggs laid by each queen was counted using a binocular microscope. One measurement was obtained for each queen, so each data point reflects an independent measurement.

OI values were estimated in both invasive and native queens by using the *length method* described in [48, 50]. In brief, this method consists in determining the relative average length of the six longest oocytes present in the ovaries to create an ovarian index for each female [48, 50]. Queens were dissected in saline solution, and the measurements were performed using ImageJ software (NIH; http://rsb.info.nih.gov/ij). Queen reproductive status was also confirmed by noting the presence or absence of sperm in the spermathecal (an opaque spermatheca indicates that a queen is mated and a translucent spermatheca indicates that a queen is virgin).

### Chemical analyses

The queens were killed by freezing (-20°C). Their gasters were then eliminated to prevent any contamination by the Dufour gland. The samples were thus composed of just their heads, thoraces, and legs. We immersed the corpses in 50 μl of dichloromethane (GC grade) during 24 hours. After 24 hours, the body parts were removed, and the resulting extract was stored at -20°C until further analysis. According to Young and Schal [51], whole body extraction allows removal CHCs without removing hydrocarbons from the interior of the insect. The extracts were evaporated and then redissolved in 50 μl of dichloromethane containing eicosane (8 ng/μl) as an internal standard. Next, 1 μl of each extract was injected into a gas chromatography (GC; Agilent 6850 Series, Agilent Technologies, USA) equipped with a flame ionisation detector and a capillary column (coated with methyl siloxane, length of 30 m, ID of 0.32 mm, film thickness of 0.25 μm; Agilent HP-1, Agilent Technologies, USA). The temperature was kept at 150°C during the initial 2 minutes of splitless injection, raised from 150°C to 300°C at 5°C/min, and held at 300°C for the last 10 minutes.

CHCs were identified using a Perkin-Meyer GC-MS functioning at 70eV with a source temperature of 230°C; the GC-MS was equipped with a ZB-5HT column (30m L x 0.25mm ID x 0.252 μm df; 5% phenyl/95% dimethylpolysiloxane). The temperature program was the same. The gas vector was helium at 2.0 ml min^-1^. CHCs were identified using standard alkanes, compound libraries, and Kovats retention indices as well as via comparison with published data [16, 26, 29, 40, 41, 52].

CHC quantities were determined using the eicosane area as the internal standard (ng per head and thorax [i.e., one queen]).

### Statistical analyses

Multivariate analyses were conducted using the relative contribution of each peak to the total. Only identified peaks whose mean relative quantities were higher than 1% in at least two groups of queens were used. To determine the degree of similarity among CHC profiles based on reproductive status and time of sampling during the execution period (before, during, and after), hierarchical cluster analysis (Euclidean distances, Ward’s method) was used to construct a single-linkage dendrogram. To identify the CHCs that characterised each group, principal component analysis (PCA) was used. We focused on the CHCs demonstrating the greatest differences—those whose factor loadings on the first axis had an absolute value > 0.75.

Discriminant analyses were also performed to investigate how CHC profiles differed based on season (winter, spring, and autumn), range (native vs. introduced), and proximity to the invasion front (contact zone vs. invaded zone). To identify the CHCs that contributed most to the differences between the groups, correlations between each CHC and the first discriminant axis were used. All the variables used in the discriminant analyses and the PCA were normally distributed after logarithmic transformation. In addition, the homogeneity of variance of these variables was tested with Levene’s test, and only those with homogeneity of variance were considered.

Generalised linear models (GLMs) were performed to compare egg-laying rates (Poisson error distribution and log–link function) and OI values (Gaussian error distribution and identity link function) based on season and reproductive status. For invasive queens, relative (%) and absolute quantities (ng/queen) of the CHCs that contributed most to the differences associated with reproductive status, fertility, and period of queen execution were compared using GLMs (relative quantities: binomial error distribution and logit link function; absolute quantities: Gaussian error distribution and identity function). When overall significant differences were detected, pairwise comparisons were performed using t-tests with pooled standard deviation; Bonferroni corrections were applied.

Finally, the ability of CHCs to function as fertility signals was investigated by looking at the correlations between the relative quantities of the CHCs that most contributed to the PCA and both the egg-laying rates and the OI values. Spearman’s rank tests were employed.

All statistical analyses were performed using R (v. 3.0.2; R Development Core Team, 2013) and Statistica (v. 6.0; StatSoft, Tulsa, OK, USA).

## Results

### CHC profiles, reproductive status, and fertility

A total of 53 compounds were detected from Argentine ant queen extracts, from which 46 were identified as hydrocarbons. Of the 46 CHCs identified, 26 met the statistical requirements for PCA and DA and were included in the statistical analyses.

**Fig 1:**
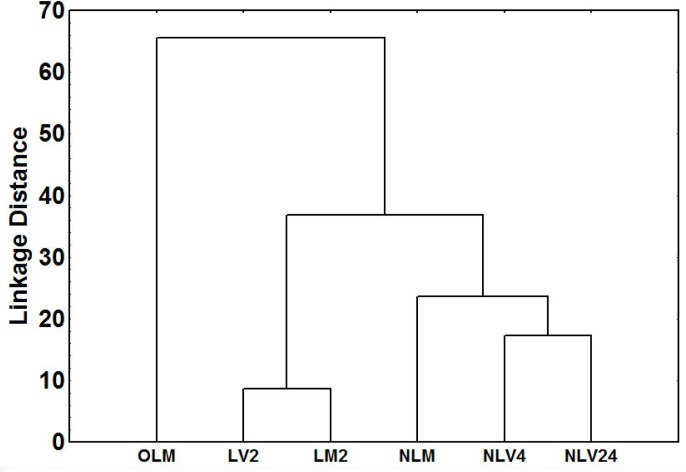
Dendrogram of chemical distances among different groups of invasive queens (Euclidean distances, Ward’s method). The abbreviations are as follows: older, field-collected queens (OLM) (n = 17), two-week-old laying mated queens (LM2) (n = 10), two-week-old laying virgin queens (LV2) (n = 9), non-laying mated queens (NLM) (n = 12), 4-day-old non-laying virgin queens (NLV4) (n = 15), and 24-hour-old non-laying virgin queens (NLV24) (n = 13).

**Fig 2:**
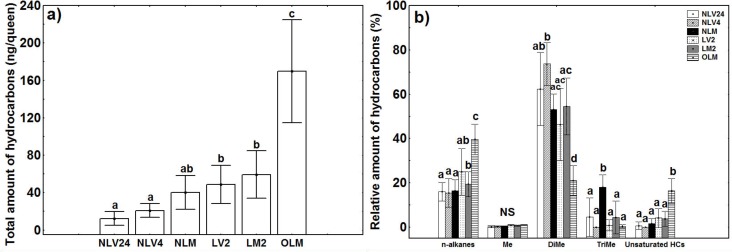
**Total absolute amount of hydrocarbons (a) and relative amount of hydrocarbons (b) in invasive queens according to reproductive status (mean ± SD) (n = 76).** The abbreviations for reproductive status are as in Fig 1. The following additional abbreviations were used in (b): mono-methyl alkanes (Me), di-methyl alkanes (DiMe), and tri-methyl alkanes (TriMe).

The cluster analysis revealed differences in chemical distances between lab-reared queens, which were younger (up to two weeks old), and field-collected queens, which were older (approximately 1 year old) (Fig 1). In addition, the total quantity of CHCs extracted from younger and older queens differed significantly with age and reproductive status (GLM: t = 6.915, *p*<0.001; results of paired t tests after Bonferroni correction: all groups of lab-reared queens (young) vs older, field collected queens—p < 0.001) (Fig 2A). In general, older queens had more linear, unsaturated alkanes and fewer di-methyl alkanes than did younger queens (GLM_linear_: t = 7.335, *p*<0.001; results of paired t tests after Bonferroni correction: all groups of lab-reared queens (young) vs older, field collected queens—p < 0.001; GLM_unsaturated_: t = 6.774, *p*<0.001; results of paired t tests after Bonferroni correction: all groups of lab-reared queens (young) vs older, field collected queens—p < 0.001; GLM_di-methyl_: t = -4.724, *p*<0.001; results of paired t tests after Bonferroni correction: all groups of lab-reared queens (young) vs older, field collected queens—p < 0.001) (Fig 2B). Three CHCs contributed the most to this difference: C_27_, C_29_, and 5,11-diMeC_35_ (S1 Table). The relative and absolute quantities of the two linear alkanes were greater in the older queens than in the younger queens (C_27_: 17.52% vs. 0.57%, GLM_relative_: *t* = 9.192, *p* < 0.001; GLM_absolute_: *t* = 7.230, *p* < 0.001; C_29_: 13.63% vs. 3.28%, GLM_relative_: *t* = 5.565, *p* < 0.001; GLM_absolute_: *t* = 3.026, *p* < 0.001). In contrast, the quantities of the di-methyl alkane were much lower in the older queens than in the younger queens (2.90% vs. 60.08%, GLM_relative_: *t* = -5.283, *p* < 0.001; GLM_absolute_: *t* = 2.684, *p* < 0.01) (S1 Table).

The cluster analysis also revealed that, although non-laying mated queens were chemically similar to non-laying virgin queens, their profiles had some differences (Fig 1). The tri-methyl alkane 5,13,15-triMeC_33_ was responsible for these differences: its relative and absolute quantitites were higher in non-laying mated queens (GLM_relative_: *t* = -5.041, *p* < 0.001; GLM_absolute_: *t* = -7.770, *p* < 0.001; results of paired *t* tests after Bonferroni correction: 24-hour-old non-laying virgin queens vs. 4-day-old non-laying virgin queens—*p* = 1; 24-hour-old and 4-day-old non-laying virgin queens vs. non-laying mated queens—*p* < 0.001) (S1 Table).

CHC profiles were also correlated with egg-laying status: laying queens were chemically distinct from non-laying queens (Fig 1). Five compounds were largely responsible for this difference. The alkene C_29:1_ (both isomers), the mono-methyl alkane 5-MeC_29_, and the di-methyl alkanes 5,11-diMeC_29_ and 5,11-diMeC_31_ represented over 1% of the CHCs found only in egg-laying queens. Furthermore, the mono-methyl alkane 5-MeC_27_ was present in egg-laying queens but only exceeded 1% in field-collected queens (i.e., older) queens (S1 Table).

Given that the quantities of these five compounds differed between laying and non-laying queens, we tried to determine if they were associated with queen fertility. We looked at the correlations between relative compound quantities and both egg-laying rates (from both laying and non-laying queens) and OI values. We found that all five hydrocarbons were positively correlated with the two variables (we only looked at the second C_29:1_ isomer in the analyses because it is the more abundant of the two) (egg-laying rate—C_29:1_: Spearman’s *r* = 0.690, *p* < 0.001; 5-MeC_27_: Spearman’s *r* = 0.486, *p* < 0.001; 5-MeC_29_: Spearman’s *r* = 0.420, *p* < 0.001; 5,11-diMeC_29_: Spearman’s *r* = 0.603, *p* < 0.001; and 5,11-diMeC_31_: Spearman’s *r* = 0.649, *p* < 0.001; OI—C_29:1_: Spearman’s *r* = 0.737, *p* < 0.001; 5-MeC_27_: Spearman’s *r* = 0.725, *p* < 0.001; 5-MeC_29_: Spearman’*s r* = 0.550, *p* < 0.001; 5,11-diMeC_29_: Spearman’s *r* = 0.629, *p* < 0.001; and 5,11-diMeC_31_: Spearman’s *r* = 0.735, *p* < 0.001).

**Fig 3:**
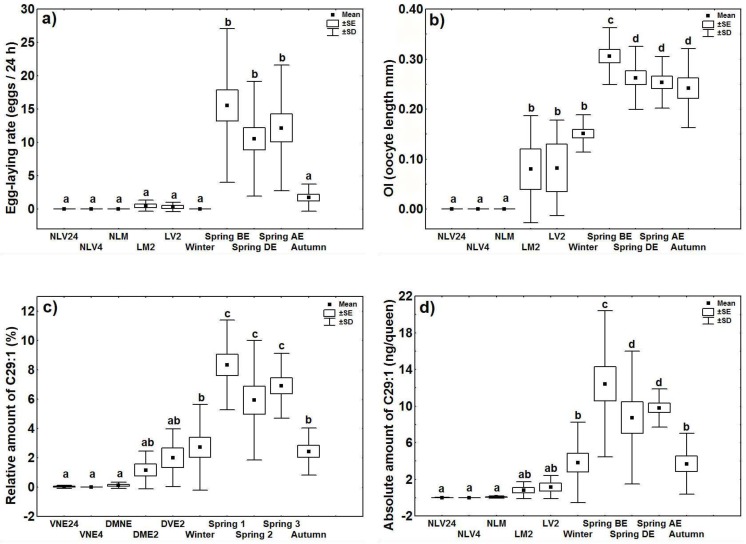
Fertility and CHC quantities in invasive queens according to reproductive status and season. **(a) egg-laying rate; (b) ovarian index (OI); (c) relative amount of C**_**29:1**_**; and (d) absolute amount of C**_**29:1**_**. All queens were fertilized except those marked as virgins.** The abbreviations for reproductive status are as in Fig 1. The following additional abbreviations were used: queens collected in February (Winter) (n = 19); queens collected before the execution period (Spring BE) (n = 17); queens collected during the execution period (Spring DE) (n = 18); queens collected after the execution period (Spring AE) (n = 17); and queens collected in September (Autumn) (n = 15).

The relative and absolute quantities of these five CHCs were then the subject of more in-depth analyses. The alkene C_29:1_ showed the highest degree of correlation with fertility. Overall, quantities of this compound differed significantly based on reproductive status and season (GLM_relative_: *t* = 8.305, *p* < 0.001; GLM_absolute_: *t* = 7.368, *p* < 0.001), which made sense given that egg-laying rate and OI also differed based on these two factors (GLM_egg-laying rate_: *t* = 5.884, *p* < 0.001; GLM_OI_: *t* = -4.393, *p* < 0.001) (Fig 3).

However, the other four CHCs also differed significantly based on reproductive status (5-MeC_27_: GLM_relative_: *t* = 8.720, *p* < 0.001; GLM_absolute_: *t* = 10.478, *p* < 0.001; 5-MeC_29_: GLM_relative_: *t* = 5.580, *p* < 0.001; GLM_absolute_: *t* = 7.297, *p* < 0.001; 5,11-diMeC_29_: GLM_relative_: *t* = 4.971, *p* < 0.001; GLM_absolute_: *t* = 7.905, *p* < 0.001; 5,11-diMeC_31_: GLM_relative_: *t* = 8.870, *p* < 0.001; GLM_absolute_: *t* = 10.996, *p* < 0.001).

Letting ourselves be guided by the previous results, we then looked at the native queens and the correlation between their OI values and the relative quantities of the five CHCs associated with fertility in invasive queens. We found positive correlations in three cases (C_29:1_: Spearman’s *r* = 0.513, *p* < 0.001; 5-MeC_27_: Spearman’s *r* = 0.622, *p* < 0.001; 5,11-diMeC_31_: Spearman’s *r* = 0.715, *p* < 0.001).

### Seasonal changes in fertility and CHC profiles

Although invasive queens did not lay eggs in the winter, their ovaries were still active. In the spring, egg-laying rates and OI values peaked. In contrast, in the autumn, egg-laying rates decreased even though queens had high OI values (Fig 3A and 3B).

The discriminant analysis revealed that CHC profiles did not differ between queens from the contact versus invaded zones; because these two groups clustered together across all seasons (*p* > 0.05 in all cases), we pooled their data in subsequent analyses. In contrast, discriminant analysis revealed seasonal differences in the CHC profiles of both native and invasive queens. In the invasive queens, two discriminant functions explained the majority of the variance (global model: Wilk’s *λ* = 0.030, *F* = 5.82, *p* < 0.001); 88.23% correct classification; first function: *R* = 0.877, Wilk’s *λ* = 0.103, *χ*^*2*^_42_ = 86.14, *p* < 0.001; second function: *R* = 0.0.742, Wilk’s *λ* = 0.450, *χ*^*2*^_20_ = 30.36, *p* < 0.005). The CHCs responsible for these differences were three di-methyl alkanes (5,11-diMeC_27;_ 5,11-diMeC_29_ and 8,10-diMeC_32_) and a single alkene (C_29:1 isomer b_) (S2 Table).

Native queens could also be discriminated based on season (global model: Wilk’s *λ* = 0.930, *F* = 5.19, *p* < 0.001; 90.16% correct classification; first function: *R* = 0.877, Wilk’s *λ* = 0.093, *χ*^*2*^_36_ = 117.590, *p* < 0.001; second function: *R* = 0.773, Wilk’s *λ* = 0.402, *χ*^*2*^_17_ = 45.14, *p* < 0.001). The CHCs responsible for these differences were two lineal alkanes (*n*-C_27_ and *n*-C_31_), a single alkene (C_29:1 isomer b_), a mono-methyl alkane (11-MeC_29_) and two di-methyl-branched alkanes (5,11-diMeC_31_ and 8,12-diMeC_32_) (S2 Table).

### CHC profiles of native and invasive queens

**Fig 4:**
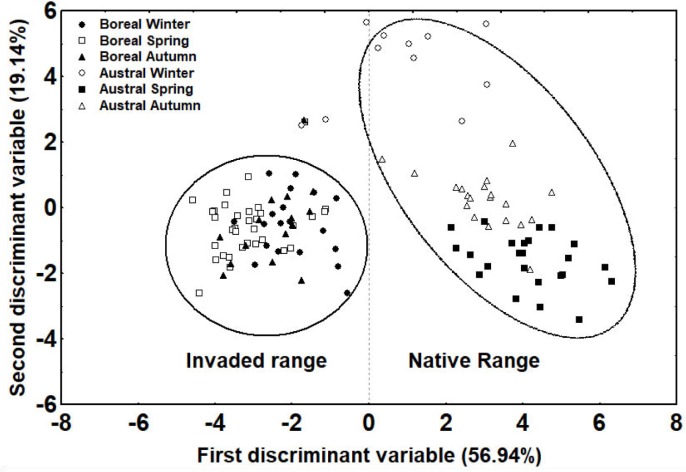
Discriminant analysis conducted using the relative quantities of CHCs in the profiles of native and invasive Argentine ant queens across seasons (winter, spring, and autumn) (n = 112).

There were significant differences in the CHC profiles of native queens versus invasive queens (Fig 4). The first discriminant function fully differentiated between them: native queens were clustered at one end of the spectrum and invasive queens were clustered at the other (89.92% correct classification; first function: *R* = 0.935, Wilk’s *λ* = 0.005, *χ*^*2*^_125_ = 594.31, *p* < 0.001). Relative quantities of a di-methyl alkane (8,12-diMeC_32_) were higher in native queens. In invasive queens, this compound was completely absent or present in only trace quantities. In addition, invasive queens had higher relative quantities of a linear alkane (C_27_) and both isomers of an alkene (C_29:1_) (S2 Table).

### CHC profiles in the post-execution period

**Fig 5:**
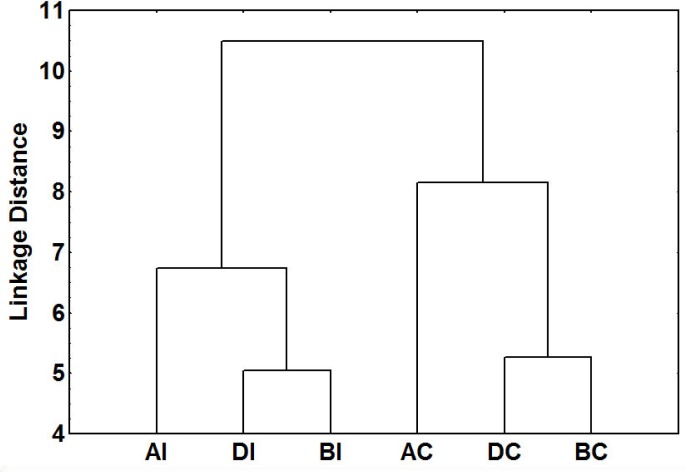
Dendrogram of chemical distances (Euclidean distances, Ward’s method) between queens collected before the execution period in the invaded zone (BI) (n = 9) and the contact zone (BC) (n = 8); during the execution period in the invaded zone (DI) (n = 8) and the contact zone (DC) (n = 10); and after the execution period in the invaded zone (AI) (n = 8) and the contact zone (AC) (n = 9).

The cluster analysis showed that the queens that survived the execution period had different CHC profiles from the queens that did not (i.e., present before and during that period). The same pattern was observed in both the contact and invaded zones (Fig 5). The main hydrocarbons responsible for these differences were three long-chained di-methyl alkanes—5,11-diMeC_29_, 5,11-diMeC_31_, and 5,11-diMeC_33_—which occurred in higher relative quantities in the surviving queens (GLM_5,11-diMeC29_: *t* = 2.82, *p* < 0.05; GLM_5,11-diMeC31_: *t* = 2.45, *p*< 0.05; GLM_5,11-diMeC33_: *t* = 2.61, *p* < 0.05; results of paired *t* tests after Bonferroni correction: queens present before vs. during execution period—*p* = 1; queens present before/during execution period vs. after execution period—*p* < 0.05) (S2 Table). The absolute quantities of these CHCs were also higher in survivors (GLM_5,11-diMeC29_: *t* = 2.30, *p* < 0.05; GLM_5,11-diMeC31_: *t* = 2.21, *p* < 0.05; GLM_5,11-diMeC33_: *t* = 2.32, *p* < 0.05; results of paired *t* tests after Bonferroni correction: queens present before vs. during execution period—*p* = 1; queens present before/during execution period vs. after execution period—*p* < 0.05).

## Discussion

The CHC profiles of Argentine ant queens displayed a complex mixture of linear, methyl-branched, di methyl-branched, and tri-methyl-branched alkanes, as well as some alkenes and one alkadiene; these CHCs ranged from C25 to C36, a wider range than reported in previous studies [41, 42]. De Biseau and colleagues [42] found CHCs between C27 and C34 in Argentine ant queens collected in southern France, whereas Vásquez and colleagues [41] found CHCs between C27 and C35 in queens from the southeastern US. We therefore discovered the presence of CHCs of both higher and lower molecular weight in queens from the main European supercolony. This finding may be simply due to the precision of measures that allowed us to find more substances.

The results of this study provide new evidence that CHCs serve as queen signals in the Argentine ant, a species with completely sterile workers, supporting the idea that CHCs do more than just regulate worker reproduction. Thus, Argentine ant queens may use CHCs to convey information about their age, reproductive status, and fertility. Indeed, in their CHC profiles, younger queens differed both qualitatively and quantitatively from older queens. These results agree with those of de Biseau and colleagues [42], who also found age-related and reproductive-status-related differences in the CHC profiles of Argentine ant queens. However, they did not identify the main compounds responsible for these differences. We discovered that younger, lab-reared queens had greater relative and absolute quantities of 5,11-diMeC_35_, regardless of reproductive status or fertility; older, field-collected queens had greater relative and absolute quantities of C_27_ and C_29_. Age-related changes in CHCs have previously been observed in other ant species [52–56].

Moreover, there was also a general increase in total CHC quantities after a queen mates [57]. Cuvillier-Hot and colleagues [54] attributed these changes to a hardening of the cuticle upon maturation, whereas Hora and colleagues [58] hypothesised that abundant CHCs provide mated queens with additional protection against abiotic factors that can increase rates of water loss, such as high temperatures or exposure to soil particles. Another non-exclusive explanation advanced by these authors is that these chemical changes provide odour cues to workers that allow them to discriminate between virgin (younger) and mated/fertile (older) queens [58]. Indeed, in the monogynous ant *Aphaenogaster senilis*, chemical differences between virgin and mated queens of the same age appear to be associated with physiological changes related to mating and egg laying; such differences could signal both caste and reproductive status [59]. In the Argentine ant, the annual execution period takes place just before the new wave of sexuals appears [20], implying that the queens in the nest at any given point in time are always rather homogenous in age. However, nothing is known about the longevity of the queens that survive execution. That small contingent coexists with the new queens for an undetermined period of time. Under these circumstances, it may be helpful to workers to be able to distinguish between younger and older queens so they can properly regulate the colony’s reproductive efficiency.

The CHC profiles of Argentine ant queens are also correlated with reproductive status. Recently mated queens (<24 hours) have undeveloped ovaries; thus, they are no more able to lay eggs than are virgin queens. However, the profiles of non-laying virgin queens and non-laying recently mated queens were different. This result suggests that workers should theoretically be able to distinguish virgin queens from recently mated queens based on chemical signatures, as has been reported in the queenless ant *Streblognathus peetersi* [60].

Interestingly, once the queens started to lay eggs, their CHC profiles converged—virgin egg layers and mated egg layers of the same age may be chemically indistinguishable to workers. Similar results have been found for the queenless ant *Dinoponera quadriceps*, in which a multivariate analysis of all the CHCs detected could not separate out virgin and mated alphas [53]. In such situations, it is possible that fertility signalling overrides the chemical cues associated with reproductive status, masking differences between virgin egg layers and mated egg layers.

Fertility could also clearly be conveyed by the CHC profiles, as both egg-laying rates and OI values were correlated with relative CHC quantities. Although the relationship between ovarian activity and CHC profiles in Argentine ant queens was assessed by de Biseau and colleagues [42], our study is the first, to our knowledge, to show a correlation between egg laying and CHC profiles in this species. We found that the main CHCs associated with egg-laying rates and OI values were an alkene (C_29:1_), two mono-methyl alkanes (5-MeC_27_ and 5-MeC_29_), and two di-methyl alkanes (5,11-diMeC_29_ and 5,11-diMeC_31_). Quantities of all five were positively correlated with egg-laying rate and OI values in invasive queens. Quantities of three of the five (C_29:1_, 5-MeC_27_, and 5,11-diMeC_31_) were correlated with OI values in native queens. These results suggest that these CHCs serve as fertility signals. Furthermore, neither the di-methyl alkane 5,11-diMeC_31_ nor the alkene C_29:1_ have been observed in the CHCs of workers from the main European supercolony [15]. In addition, the alkene C_29:1_—which showed the highest degree of correlation with fertility—was completely absent in workers from other Argentine ant supercolonies, namely the Corsican, the Catalan, the Californian, the Japanese, the South African, and the Australian supercolonies [15, 29, 31, 32, 37, 52]. This finding supports the idea that this particular CHC is characteristic of the Argentine ant queen’s CHC profile (as suggested by de Biseau and colleagues [42]) and thus plays an important role in signalling fertility. These results agree with those of previous studies that found fertility could be signalled by a blend of alkenes, branched alkenes, and/or long-chain hydrocarbons (reviewed in [6]).

We also observed seasonal differences in the CHC profiles of both native and invasive queens. Studies in other ant species have previously observed seasonal CHC shifts in workers [61–63], and Liu and colleagues [63] hypothesised that these changes were the result of individuals responding to seasonal environmental variation (e.g., in temperature) that affects cuticular lipid composition. Our finding that the CHC profiles of queens also varied seasonally is novel. Given that queens are reproductives, another non-exclusive explanation for these changes is that they are induced by seasonal variation in queen fertility. Indeed, in the winter, the queens stopped laying, mainly due to their lower OI values. In the spring, however, they began laying eggs again, and egg-laying rates and OI values peaked. These results fit with observations on the species’ seasonal life cycle [18, 19]. However, unlike what has been described elsewhere based on observations of nest brood, we did not see a resurgence in egg laying in the autumn, even though OI values were high. Since egg laying in this species is strongly affected by temperature [48], these results were likely related to the decline in nest temperatures during this period.

Native queens (taken from the Otamendi Reserve) and invasive queens (taken from the main European supercolony) differed in their CHC profiles, namely in their relative quantities of different hydrocarbons. Within species, nestmate recognition is based on quantitative rather than qualitative differences in profiles [36, 64]. Previous studies have suggested that, in the Argentine ant, nestmate recognition cues rely on mixtures of CHCs of different structural classes rather than on a few compounds of a single structural class [28, 40, 41]. These results, plus our finding that a mixture of structurally different CHCs (*n*-alkanes, di-methyl alkanes, and one alkene) distinguished native from invasive queens, suggest that these CHCs may chemically identify queens from these two populations. However, behavioural studies testing the responses elicited by the various CHCs identified in our study should be conducted to determine how these compounds influence queen recognition.

That said, the genetic drift associated with the founding event that resulted in the main European population does not seem to have qualitatively affected profile composition because the native and invasive queens had similar CHC profiles. In addition, our findings indicate that the queens also shared certain CHC correlated with ovarian activity, suggesting that fertility signalling is similar despite the loss of genetic and chemical diversity in invasive populations [30].

Our results also provide new insight into queen executions in the Argentine ant. The fact that surviving queens had different CHC profiles from queens present before and during the execution period suggests that workers may cull queens based on their CHC profiles. Surviving queens had greater quantities of several di-methyl alkanes, two of which were also correlated with egg-laying rate and OI value. These CHCs could also convey information about a queen’s overall condition or her competitive abilities, as seen in *Lasius niger* [56, 65]. In this latter species, pleometrosis (i.e., colony founding by multiple queens) ends with the workers executing all but one queen. Queens appear to be marked for execution because of lower productivity since several CHCs that have been associated with reproductive maturity and productivity occur in greater quantities in surviving queens [56].

In conclusion, this study highlights the complexity of queen signalling in a highly polygynous ant species with permanently sterile workers. We identified several chemical compounds present in the queen’s cuticle that are correlated with age, reproductive status, fertility, and queen survival. Although the relationship between the CHCs in Argentine ant queens and their ovarian activity has been investigated before [42], to our knowledge, this is the first time that a study provides information about the specific compounds involved in the communication of age, reproductive status and fertility in this species. Moreover, our results also provide for the first time information about the CHC composition of Argentine ant queens from their native range in relation to their physiological status, and also about the seasonal dynamics of CHC for queens in both native and introduced ranges. In addition, we yield new insights into the Argentine ant queen executions, since our finding that variations in cuticular hydrocarbons can be the basis of queen executions in this species is novel.

On the other hand, the results obtained here support the “queen signal” hypothesis [1, 3, 4] and suggest an additional perspective on the biological function of CHCs in queens. Indeed, their purpose appears to extend beyond the repression of worker reproduction.

## Supporting information

S1 Table Relative quantities (%) of cuticular hydrocarbons (CHCs) in younger, lab-reared queens and older, field-sampled queens from the Argentine ant’s introduced range (mean ± SE). The text in bold indicates the CHCs that differed most among the groups. The peaks marked with an asterisk indicate the CHCs included in the statistical analyses.
(DOCX)

S2 Table Relative quantities (%) of cuticular hydrocarbons (CHCs) across seasons in field-sampled queens from the Argentine ant’s native and introduced ranges (mean ± SE). The text in bold indicates the CHCs that differed most among the groups. The peaks marked with an asterisk indicate the compounds included in the statistical analyses.
(DOCX)

S1 File Raw data obtained in the chemical analyses. Numbers in bold correspond to each of the 46 CHCs identified from the Argentine ant extracts, and the values correspond to its area.
(XLSX)
