# Supplementary material for: Cuticular hydrocarbons correlate with queen reproductive status in native and invasive Argentine ants (Linepithema humile, Mayr)
Source: PLoS One. 2018 Feb 22;13(2):e0193115. doi: 10.1371/journal.pone.0193115 (PMC5823440; doi:10.1371/journal.pone.0193115)
Supplement: S1 Table — The text in bold indicates the CHCs that differed most among the groups. The peaks marked with an asterisk indicate the CHCs included in the statistical analyses. (DOCX) [file pone.0193115.s001.docx]

**S1 Table. Relative quantities (%) of cuticular hydrocarbons (CHCs) in younger, lab-reared queens and older, field-sampled queens from the Argentine ant’s introduced range (mean ± SE).** The text in bold indicates the CHCs that differed most among the groups. The peaks marked with an asterisk indicate the CHCs included in the statistical analyses.

| Peak number | Compound | 24-hour-old non-laying virgin queens  (n=13) | 4-day-old non-laying virgin queens  (n=15) | Non-laying mated queens  (n=12) | 2-week-old laying mated queens  (n=10) | 2-week-old laying virgin queens  (n=9) | Older, field-collected queens in spring (April-approx. 1 year old)  (n=17) |
| --- | --- | --- | --- | --- | --- | --- | --- |
| 1* | *n*-C_25_ | 10.47 ± 1.3 | 4.68 ± 0.67 | 2.57 ± 0.32 | 1.25 ± 0.39 | 1.79 ± 0.42 | 2.16 ± 0.26 |
| 2* | 5-MeC_25_ | - | - | - | - | - | 0.45 ± 0.10 |
| 3* | *n*-C_26_ | - | - | - | - | - | 0.49 ± 0.08 |
| 4a | xC_27:1_ | - | - | - | - | - | 0.64 ± 0.15 |
| 4b* | xC_27:1_ | 0.15 ± 0.15 | - | - | - | - | 2.03 ± 0.52 |
| **5*** | ***n*-C_27_** | **0.57 ± 0.33** | **2.84 ± 0.58** | **2.44 ± 0.30** | **4.20 ± 0.54** | **5.49 ± 1.21** | **17.52 ± 0.98** |
| 6 | 11-MeC_27_ | - | - | - | - | - | 0.26 ± 0.09 |
| 7 | 7-MeC_27_ | - | - | - | - | - | - |
| **8*** | **5-MeC_27_** | **-** | **-** | **-** | **0.36 ± 0.15** | **0.61 ± 0.23** | **3.18 ± 0.26** |
| 9 | 3-MeC_27_ | 0.19 ± 0.13 | 0.11 ± 0.11 | 0.30 ± 0.13 | 1.01 ± 0.37 | 1.11 ± 0.33 | 0.52 ± 0.12 |
| 10* | 5,11-diMeC_27_ | - | - | - | - | 0.15 ± 0.11 | 1.16 ± 0.16 |
| 11* | *n*-C_28_ | - | - | 0.19 ± 0.08 | 0.57 ± 0.21 | 0.78 ± 0.27 | 0.63 ± 0.11 |
| 12* | 8+10-MeC_28_ | - | - | - | - | 0.32 ± 0.22 | 0.76 ± 0.14 |
| 13 | 4-MeC_28_ | - | - | - | - | - | - |
| **14a*** | **xC_29:1_** | **-** | **-** | **-** | **1.52 ± 0.44** | **1.12 ± 0.42** | **3.46 ± 0.45** |
| **14b*** | **xC_29:1_** | **-** | **-** | **0.11 ± 0.06** | **1.16 ± 0.40** | **2.00 ± 0.65** | **8.33 ± 0.74** |
| 15 | 4,10-diMeC_28_ | - | - | - | - | - | - |
| **16*** | ***n*-C_29_** | **3.28 ± 0.84** | **5.63 ± 0.80** | **4.73 ± 0.53** | **9.13 ± 0.98** | **10.34 ± 2.06** | **13.63 ± 0.94** |
| 17* | 11-MeC_29_ | - | - | 0.24 ± 0.12 | 1.70 ± 0.59 | 2.16 ± 0.83 | 1.71 ± 0.25 |
| 18 | 7-MeC_29_ | 1.31 ± 0.52 | 1.29 ± 0.43 | 0.67 ± 0.20 | 0.71 ± 0.28 | 0.96 ± 0.28 | 0.13 ± 0.12 |
| **19*** | **5-MeC_29_** | **-** | **-** | **0.30 ± 0.14** | **3.33 ± 0.81** | **3.20 ± 1.02** | **4.45 ± 0.72** |
| 20* | 3-MeC_29_ | 0.18 ± 0.12 | 1.52 ± 0.38 | 1.60 ± 0.30 | 2.00 ± 0.42 | 2.56 ± 0.45 | 1.08 ± 0.10 |
| **21*** | **5,11-diMeC_29_** | **-** | **-** | **0.35 ± 0.16** | **6.24 ± 1.90** | **6.00 ± 2.38** | **6.00 ± 0.72** |
| 22 | *n*-C_30_ | - | - | - | - | 0.22 ± 0.11 | - |
| 23 | 8+10-MeC_30_ | 0.17 ± 0.17 | - | - | - | - | 0.97 ± 0.34 |
| 24 | 8,12-diMeC_30_ | - | - | - | - | - | 0.48 ± 0.14 |
| 25 | xC_31:1_ | - | - | 0.18 ± 0.07 | - | 0.24 ± 0.20 | 0.21 ± 0.21 |
| 26* | *n*-C_31_ | 0.60 ± 0.47 | 1.95 ± 0.95 | 1.24 ± 0.45 | 2.92 ± 0.50 | 3.60 ± 0.72 | 4.13 ± 0.43 |
| 27 | 11+13-MeC_31_ | 0.21 ± 0.22 | - | - | - | - | - |
| 28* | 15-Me C_31_ | 2.30 ± 1.07 | 5.01 ± 1.22 | 0.55 ± 0.25 | 1.87 ± 0.79 | 1.32 ± 0.91 | 1.23 ± 0.25 |
| 29* | 5-MeC_31_ | 0.11 ± 0.11 | - | 0.21 ± 0.10 | 1.15 ± 0.34 | 2.20 ± 0.60 | 2.88 ± 0.54 |
| **30*** | **5,11-diMeC_31_** | **0.21 ± 0.14** | **0.15 ± 0.15** | **0.53 ± 0.20** | **1.90 ± 0.33** | **1.40 ± 0.46** | **3.78 ± 0.55** |
| 31* | *n*-C_32_ | 0.22 ± 0.22 | 0.47 ± 0.47 | - | - | - | 0.97 ± 0.30 |
| 32 | 12+14-MeC_32_ | - | - | - | - | - | 0.96 ± 0.14 |
| 33* | 8,10-diMeC_32_ | - | - | - | - | - | 1.18 ± 0.30 |
| 35* | Unknown | 9.96 ± 2.37 | 3.43 ± 0.83 | 3.99 ± 0.66 | 2.95 ± 0.41 | 4.32 ± 0.55 | 2.82 ± 0.33 |
| 36a* | xC_33:2_ | 0.30 ± 0.21 | - | 0.52 ± 0.20 | 0.92 ± 0.39 | 0.73 ± 0.43 | 1.12 ± 0.23 |
| 36b* | xC_33:2_ | 0.22 ± 0.15 | - | 0.42 ± 0.16 | - | - | 0.58 ± 0.15 |
| 37* | 4,10-diMeC_32_ | 1.53 ± 1.45 | - | - | - | 0.17 ± 0.18 | 1.02 ± 0.39 |
| 38 | xC_33:1_ | - | - | 0.25 ± 0.20 | 0.20 ± 0.13 | - | - |
| 39 | 11-MeC_33_ | - | - | - | - | - | - |
| 40* | 5-MeC_33_ | 0.43 ± 0.31 | - | 0.26 ± 0.26 | 0.57 ± 0.30 | 0.96 ± 0.59 | 0.87 ± 0.18 |
| 41* | 5,11-diMeC_33_ | 11.18 ± 1.17 | 12.18 ± 0.63 | 5.91 ± 1.06 | 8.37 ± 0.63 | 7.38 ± 1.33 | 4.14 ± 0.55 |
| **42*** | **5,13,15-triMeC_33_** | **4.58 ± 2.42** | **-** | **18.14 ± 1.60** | **4.50 ± 2.34** | **0.83 ± 0.83** | **0.33 ± 0.19** |
| 43 | 11-MeC_35_ | - | - | - | - | - | - |
| 44* | 5-MeC_35_ | 0.69 ± 0.53 | - | 1.98 ± 1.22 | 1.90 ± 1.30 | 3.64 ± 2.10 | - |
| 45a | 5,11-diMeC_35_ | 1.44 ± 1.44 | - | - | - | - | 0.30 ± 0.22 |
| **45b*** | **5,11-diMeC_35_** | **47.79 ± 5.05** | **60.08 ± 2.37** | **46.20 ± 2.23** | **37.94 ± 3.20** | **31.18 ± 5.32** | **2.90 ± 0.63** |
| 46 | *n*-C_36_ | 0.87 ± 0.87 | 0.61 ± 0.61 | 5.19 ± 1.74 | 1.36 ± 0.70 | 2.78 ± 1.41 | - |
