# Supplementary material for: Cuticular hydrocarbons correlate with queen reproductive status in native and invasive Argentine ants (Linepithema humile, Mayr)
Source: PLoS One. 2018 Feb 22;13(2):e0193115. doi: 10.1371/journal.pone.0193115 (PMC5823440; doi:10.1371/journal.pone.0193115)
Supplement: S2 Table — The text in bold indicates the CHCs that differed most among the groups. The peaks marked with an asterisk indicate the compounds included in the statistical analyses. (DOCX) [file pone.0193115.s002.docx]

**S2 Table. Relative quantities (%) of cuticular hydrocarbons (CHCs) across seasons in field-sampled queens from the Argentine ant’s native and introduced ranges (mean ± SE).** The text in bold indicates the CHCs that differed most among the groups. The peaks marked with an asterisk indicate the compounds included in the statistical analyses.

|  |  | INTRODUCED RANGE | | | | | NATIVE RANGE | | |
| --- | --- | --- | --- | --- | --- | --- | --- | --- | --- |
|  |  |  | | | | |  | | |
| Peak number | Compound | Queens sampled in the winter  (February; n=19) | Queens sampled in the spring before the execution period (April; n=17) | Queens sampled in the spring during execution period (early May; n=18) | Queens sampled in the spring after the execution period  (end of May; n=17) | Queens sampled in the autumn  (September; n=15) | Queens sampled in the winter  (July; n=15) | Queens sampled in the spring (October and November; n=26) | Queens sampled in the autumn (April; n=20) |
| 1* | *n*-C_25_ | 2.03 ± 0.23 | 2.16 ± 0.26 | 1.95 ± 0.16 | 1.41 ± 0.18 | 1.44 ± 0.14 | 3.32 ± 0.34 | 11.10 ± 13.34 | 6.96 ± 2.58 |
| 2* | 5-MeC_25_ | 0.58 ± 0.09 | 0.45 ± 0.10 | 0.39 ± 0.07 | 0.30 ± 0.08 | 0.36 ± 0.09 | 1.05 ± 0.24 | 0.84 ± 2.05 | 3.93 ± 0.48 |
| 3* | *n*-C_26_ | 0.74 ± 0.09 | 0.49 ± 0.08 | 0.54 ± 0.07 | 0.47 ± 0.06 | 0.76 ± 0.09 | 0.91 ± 0.46 | 25.75 ± 25.61 | 13.92 ± 1.55 |
| 4a | xC_27:1_ | 0.72 ± 0.17 | 0.64 ± 0.15 | 0.49 ± 0.10 | 0.30 ± 0.08 | - | 0.10 ± 0.05 | - | - |
| 4b* | xC_27:1_ | 0.71 ± 0.28 | 2.03 ± 0.52 | 3.34 ± 0.65 | 2.54 ± 0.34 | 1.32 ± 0.26 | 1.10 ± 0.31 | 0.32 ± 1.43 | 2.03 ± 0.43 |
| **5*** | ***n*-C_27_** | **22.58 ± 2.48** | **17.52 ± 0.98** | **19.05 ± 1.13** | **14.40 ± 1.02** | **21.49 ± 2.37** | **13.44 ± 2.20** | **4.12 ± 9.99** | **7.63 ± 0.98** |
| 6 | 11-MeC_27_ | 0.50 ± 0.12 | 0.26 ± 0.09 | 0.39 ± 0.1 | - | 0.52 ± 0.12 | - | 0.45 ± 1.76 | 0.87 ± 0.23 |
| 7 | 7-MeC_27_ | 0.18 ± 0.07 | - | - | - | - | 0.10 ± 0.09 | - | 0.33 ± 0.17 |
| 8* | 5-MeC_27_ | 4.60 ± 0.39 | 3.18 ± 0.26 | 3.02 ± 0.30 | 2.76 ± 0.26 | 4.15 ± 0.70 | 4.34 ± 0.66 | 0.96 ± 2.02 | 3.71 ± 0.44 |
| 9 | 3-MeC_27_ | 0.51 ± 0.10 | 0.52 ± 0.12 | 0.51 ± 0.13 | 0.30 ± 0.10 | 0.27 ± 0.09 | - | - | 0.77 ± 0.19 |
| **10*** | **5,11-diMeC_27_** | **1.09 ± 0.28** | **1.16 ± 0.16** | **1.05 ± 0.17** | **1.03 ± 0.10** | **1.42 ± 0.19** | 1.77 ± 0.28 | - | 0.65 ± 0.17 |
| 11* | *n*-C_28_ | 0.80 ± 0.20 | 0.63 ± 0.11 | 0.85 ± 0.24 | 0.52 ± 0.11 | 0.93 ± 0.19 | 0.21 ± 0.08 | 3.26 ± 6.23 | 4.47 ± 1.20 |
| 12* | 8+10-MeC_28_ | 0.69 ± 0.14 | 0.76 ± 0.14 | 0.63 ± 0.14 | 0.83 ± 0.11 | 1.31 ± 0.19 | 0.30 ± 0.16 | 4.34 ± 9.60 | - |
| 13 | 4-MeC_28_ | 0.22 ± 0.05 | - | - | - | - | - | - | - |
| **14a*** | **xC_29:1_** | **1.36 ± 0.54** | **3.46 ± 0.45** | **2.49 ± 0.50** | **2.40 ± 0.37** | **1.43 ± 0.34** | **0.50 ± 0.27** | **-** | **-** |
| **14b*** | **xC_29:1_** | **2.71 ± 0.67** | **8.33 ± 0.74** | **5.92 ± 0.96** | **6.91 ± 0.53** | **2.43 ± 0.41** | **1.55 ± 0.42** | **0.37 ± 1.00** | **1.07 ± 0.23** |
| 15 | 4,10-diMeC_28_ | 0.22 ± 0.05 | - | - | 0.31 ± 0.22 | 0.22 ± 0.07 | - | - | - |
| 16* | *n*-C_29_ | 16.29 ± 1.85 | 13.63 ± 0.94 | 15.70 ± 1.28 | 14.88 ± 1.03 | 19.04 ± 1.50 | 5.65 ± 1.37 | 9.68 ± 13.05 | 10.07 ± 1.35 |
| 17* | 11-MeC_29_ | 1.64 ± 0.36 | 1.71 ± 0.25 | 1.21 ± 0.32 | 2.05 ± 0.27 | 3.11 ± 0.48 | 2.37 ± 0.32 | 0.2 ± 1.05 | 1.88 ± 0.34 |
| 18* | 7-MeC_29_ | 0.74 ± 0.27 | 0.13 ± 0.12 | 0.69 ± 0.26 | 0.18 ± 0.16 | - | 0.22 ± 0.21 | - | - |
| 19* | 5-MeC_29_ | 2.95 ± 0.77 | 4.45 ± 0.72 | 3.58 ± 0.85 | 5.79 ± 0.90 | 3.99 ± 0.86 | 2.28 ± 0.90 | 5.59 ± 8.49 | 3.90 ± 0.44 |
| 20* | 3-MeC_29_ | 0.73 ± 0.16 | 1.08 ± 0.10 | 0.88 ± 0.12 | 1.21 ± 0.13 | 0.56 ± 0.15 | 0.18 ± 0.10 | 0.16 ± 0.86 | 2.93 ± 2.37 |
| **21*** | **5,11-diMeC_29_** | 4.41 ± 2.00 | **6.00 ± 0.72** | 5.99 ± 0.94 | **8.29 ± 0.94** | 8.68 ± 1.70 | 4.54 ± 1.67 | 10.26 ± 15.05 | 9.15 ± 2.01 |
| 22 | *n*-C_30_ | 0.28 ± 0.07 | - | - | - | 0.25 ± 0.08 | 0.13 ± 0.08 | 0.54 ± 1.83 | 2.04 ± 0.55 |
| 23 | 8+10-MeC_30_ | 0.51 ± 0.17 | 0.97 ± 0.34 | 0.41 ± 0.15 | 0.44 ± 0.22 | 0.49 ± 0.20 | 0.84 ± 0.32 | - | 0.21 ± 0.10 |
| 24 | 8,12-diMeC_30_ | 0.18 ± 0.06 | 0.48 ± 0.14 | 0.19 ± 0.09 | 0.11 ± 0.06 | - | 3.09 ± 0.85 | - | - |
| 25 | xC_31:1_ | 0.20 ± 0.08 | 0.21 ± 0.21 | 0.65 ± 0.37 | - | 0.19 ± 0.16 | - | 0.41 ± 1.89 | - |
| **26*** | ***n*-C_31_** | 4.14 ± 0.98 | 4.13 ± 0.43 | 3.89 ± 0.51 | 5.07± 0.42 | 3.33 ± 0.76 | **7.21 ± 1.28** | **1.65 ± 3.42** | **4.50 ± 0.82** |
| 27 | 11+13-MeC_31_ | - | - | - | - | 0.15 ± 0.14 | - | - | - |
| 28* | 15-MeC_31_ | 1.15 ± 0.32 | 1.23 ± 0.25 | 1.55 ± 0.26 | 1.72 ± 0.23 | 0.17 ± 0.12 | 3.73 ± 0.83 | 0.22 ± 1.13 | 0.99 ± 0.31 |
| 29* | 5-MeC_31_ | 2.74 ± 0.55 | 2.88 ± 0.54 | 2.46 ± 0.44 | 2.64 ± 0.62 | 2.82 ± 0.46 | 1.59 ± 0.81 | 7.45 ± 9.21 | 3.17 ± 0.46 |
| **30*** | **5,11-diMeC_31_** | 3.00 ± 0.64 | **3.78 ± 0.55** | 4.03 ± 0.66 | **5.36 ± 0.51** | 4.80 ± 0.59 | **8.89 ± 1.19** | **1.22 ± 2.86** | **1.84 ± 0.39** |
| 31* | *n*-C_32_ | 0.22 ± 0.16 | 0.97 ± 0.30 | 1.43 ± 0.34 | 0.94 ± 0.33 | 1.30 ± 0.26 | 0.59 ± 0.37 | 0.56 ± 1.38 | 1.45 ± 0.28 |
| 32 | 12+14-MeC_32_ | 0.41 ± 0.11 | 0.96 ± 0.14 | 0.86 ± 0.11 | 0.62 ± 0.14 | 0.55 ± 0.08 | 0.69 ± 0.25 | 0.11 ± 0.59 | 0.27 ± 0.17 |
| **33*** | **8,10-diMeC_32_** | **0.35 ± 0.11** | **1.18 ± 0.30** | **0.64 ± 0.21** | **0.26 ± 0.13** | **-** | 0.15 ± 0.15 | 0.30 ± 1.13 | 0.38 ± 0.24 |
| **34*** | **8,12-diMeC_32_** | **0.12 ± 0.04** | **-** | **-** | **0.10 ± 0.07** | **-** | **9.62 ± 0.80** | **0.99 ± 2.43** | **3.66 ± 0.61** |
| 35* | Unknown | 2.01 ± 0.31 | 2.82 ± 0.33 | 2.81 ± 0.39 | 2.27 ± 0.24 | 2.12 ± 0.24 | 4.38 ± 0.75 | 0.14 ± 0.73 | - |
| 36a* | xC_33:2_ | 0.69 ± 0.14 | 1.12 ± 0.23 | 1.22 ± 0.25 | 1.31 ± 0.18 | 0.42 ± 0.15 | 0.54 ± 0.27 | 0.92 ± 3.48 | - |
| 36b* | xC_33:2_ | 0.56 ± 0.21 | 0.58 ± 0.15 | 0.61 ± 0.18 | 0.98 ± 0.16 | 1.05 ± 0.22 | 2.84 ± 0.33 | 0.15 ± 0.76 | 0.11 ± 0.06 |
| 37* | 4,10-diMeC_32_ | 0.46 ± 0.19 | 1.02 ± 0.39 | 0.33 ± 0.14 | 0.86 ± 0.37 | 1.09 ± 0.57 | - | 0.15 ± 0.75 | 0.26 ± 0.15 |
| 38 | xC_33:1_ | 0.32 ± 0.15 | - | 0.15 ± 0.10 | - | - | 4.67 ± 1.02 | 0.73 ± 2.76 | - |
| 39 | 11-MeC_33_ | - | - | - | - | - | 2.60 ± 1.20 | - | 0.19 ± 0.14 |
| 40* | 5-MeC_33_ | 1.45 ± 0.28 | 0.87 ± 0.18 | 0.97 ± 0.19 | 1.18 ± 0.19 | 1.12 ± 0.22 | 1.72 ± 0.41 | 1.08 ± 3.62 | 1.26 ± 0.27 |
| **41*** | **5,11-diMeC_33_** | 5.42 ± 0.93 | **4.14 ± 0.55** | 4.48 ± 0.81 | **6.11 ± 0.73** | 3.77 ± 0.71 | 0.39 ± 0.39 | 3.76 ± 7.30 | 3.99 ± 0.75 |
| 42* | 5,13,15-triMeC_33_ | 1.45 ± 0.46 | 0.33 ± 0.19 | 0.51 ± 0.25 | 0.26 ± 0.12 | 0.47 ± 0.20 | 0.59 ± 0.34 | 1.62 ± 6.41 | 0.27 ± 0.18 |
| 43 | 11-MeC_35_ | 0.64 ± 0.23 | - | 0.12 ± 0.07 | 0.11 ± 0.06 | 0.44 ± 0.15 | 0.59 ± 0.36 | - | 0.51 ± 0.43 |
| 44* | 5-MeC_35_ | - | - | 0.16 ± 0.09 | - | - | 0.18 ± 0.18 | 0.18 ± 0.91 | - |
| 45a | 5,11-diMeC_35_ | 0.67 ± 0.37 | 0.30 ± 0.22 | 0.26 ± 0.26 | - | 0.10 ± 0.10 | - | - | - |
| **45b*** | **5,11-diMeC_35_** | **5.50 ± 0.93** | **2.90 ± 0.63** | **2.93 ± 0.45** | **1.98 ± 0.25** | **1.21 ± 0.40** | 0.20 ± 0.14 | **-** | **-** |
| 46 | *n*-C_36_ | 0.18 ± 0.09 | - | - | - | - | 0.24 ± 0.14 | - | - |
